# Is There a Predictable Cost-Benefit Ratio in Preeclampsia?

**DOI:** 10.7759/cureus.41051

**Published:** 2023-06-27

**Authors:** Dogukan Ozkan, Betul Tokgoz Cakir, Ceren Polat Kamaci, Merve Ozkan, Cantekin Iskender, Omer Tapisiz, Yaprak Engin-Üstün

**Affiliations:** 1 Obstetrics and Gynaecology, Etlik Zübeyde Hanim EAH, Ankara, TUR; 2 Obstetrics and Gynecology, University of Health Sciences Etlik Zübeyde Hanim Women’s Health Training and Research Hospital, Ankara, TUR; 3 Obstetrics and Gynecology, Zekai Tahir Burak Women’s Health Research and Education Hospital, Ankara, TUR

**Keywords:** aspartate aminotransferase platelet ratio index (apri), platelet lymphocyte ratio (plr), neutrophil lymphocyte ratio (nlr), severe preeclampsia, preeclampsia, gestational hypertension

## Abstract

Background

Preeclampsia (PE) is one of the highest-risk pregnancies and a complicated condition that occurs in 2% to 8% of pregnancies and is associated with markers of a systemic inflammatory response (SIR). In this study, we aimed to determine the role of these markers in predicting PE.

Methodology

A total of 300 women with singleton pregnancies and cephalic presentation were included in the study. Normotensive pregnant women (n = 149) who met this criterion were included as the control group Pregnant women who met the inclusion criteria for a diagnosis of preeclampsia (n = 151) were included in the study group.

Results

The baseline characteristics of the study groups showed no significant difference. The hypertensive group was hospitalized significantly earlier than the control group (p < 0.001). We found significantly higher systolic and diastolic blood pressure values in the PE group than in the other group (p < 0.001). The mean neutrophil-to-lymphocyte ratio (NLR), platelet-to-lymphocyte ratio (PLR), and aspartate aminotransferase-to-platelet ratio index (APRI) values at hospitalization did not differ significantly between groups (p = 0.639, p = 0.709, and p = 0.066, respectively). In the receiver operating characteristic analysis curves compared with the control group and PE, none of the parameters could predict PE.

Conclusions

We found that NLR, PLR, and APRI have no clinical significance in assessing developmental risk and predicting PE.

## Introduction

Hypertensive disorders during pregnancy can have serious adverse effects on the mother and fetus and can even lead to death [1]. Hypertensive disorders can occur suddenly in about 10% of all pregnancies in any trimester [2,3]. It is one of the leading causes of maternal mortality worldwide (16%) [3,4]. Gestational hypertension in a previously normotensive woman is defined by the American College of Obstetricians and Gynecologists (ACOG) as systolic blood pressure (SBP) ≥140 mmHg and/or diastolic blood pressure (DBP) ≥90 mmHg at the 20th week of gestation with two measurements at least four hours apart [3]. Preeclampsia (PE) is a condition that occurs after the 20th week of gestation, usually just before delivery. It is a multisystemic disease characterized by simultaneous dysfunction of various end organs with or without new-onset hypertension and concomitant new-onset proteinuria [3,5].

It appears that these inflammatory processes may be important in predicting the disease in hypertensive disorders in pregnancy, which are associated with the activation of various inflammatory processes and eventually with ischemia and vasoconstriction of the end organs [6-9]. There are many studies on this topic. However, only 30% of women who are likely to have PE can be predicted [6]. There are several studies on low-cost and reliable screening tests that can give scientists an indication before the disease occurs based on the general pathophysiology [6]. In this context, it is important to note that this disease, which has high costs for diagnosis, treatment, and management due to its impact on pregnancy and the baby, has become a preventable disease through screening tests [10,11]. However, there is still no proven effective test with high predictive value for PE [3]. The neutrophil-to-lymphocyte ratio (NLR), platelet-to-lymphocyte ratio (PLR), and aspartate aminotransferase-to-platelet ratio index (APRI) have recently gained popularity because they can be easily calculated from routine blood values and are cost-effective due to the definition of inflammatory processes as early changes [12-15]. Studies have suggested that the PLR, NLR, and APRI are useful for predicting PE [16,17].

This study aims to demonstrate the usefulness of the APRI, NLR, and PLR parameters as predictive factors for preeclampsia and to determine whether these parameters can be used as screening tests.

## Materials and methods

This cohort study included 300 pregnant women enrolled in a tertiary obstetrics research and training center between January 2021 and December 2021. This study was approved by the Institutional Review Board on December 30, 2020 (approval number: #2020/177). Signed informed consent was obtained from all participants, and the study followed the guidelines of the Declaration of Helsinki on Research Involving Human Subjects.

In this study, 151 pregnant women with pregnancy-related hypertensive disease were included in the study group and 149 normotensive pregnant women were included in the control group. The patients’ diagnoses were made according to the ACOG Pregnancy Hypertension and Preeclampsia Bulletin published in June 2020 [3]. Patients aged 18-45 years with a pregnancy at 24-40 weeks gestation, those diagnosed with gestational hypertension, PE, or severe PE, and those who were registered and delivered at our hospital were included in this study. Patients with multiple pregnancies, additional systemic and chronic diseases (liver diseases such as chronic hypertension, diabetes mellitus, cholestasis, heart diseases, rheumatic diseases, and kidney diseases), chorioamnionitis, fetal anomalies, infectious diseases, corticosteroid therapy, and who smoked seven days before delivery were excluded.

Serum and urine samples for the inflammatory parameters to be investigated in the study were collected when the patients arrived at the hospital, i.e., before the start of medical treatment, such as the administration of magnesium sulfate or antenatal corticosteroids. Venous blood samples of 10 cc were collected in tubes containing ethylenediaminetetraacetic acid (EDTA). Subsequently, all samples were centrifuged at 4°C for 10 minutes within two hours of sample collection. Complete blood count parameters were determined using the ADVIA 2120i (Siemens Healthcare), and biochemical parameters were determined using the AU680 and AU480 devices (Beckman Coulter). These instruments are not specific to this study but are commonly used for all samples in the laboratory.

Based on the results obtained, the parameters to be used in our study were determined using the following calculations: (1) APRI = (AST (IU/L)/upper limit of AST (IU/L))/patient’s platelet count (10^9^/L) × 100 [18]; (2) NLR = neutrophil count/lymphocyte count; and (3) PLR = platelet count/lymphocyte count.

Medical history and demographic data (age, pregnancy history, height and weight, and blood pressure values measured during hospitalization (systolic, diastolic)) were obtained from hospital records.

Statistical analysis

Measured values were examined and reported as means ± standard deviation (SD). Statistical analysis was performed using SPSS version 16 (IBM Corp., Armonk, NY, USA) and SAS Enterprise Guide (SAS Institute Inc., Cary, NC, USA). The normality of the distribution of the groups was juxtaposed using the Shapiro-Wilk test. Parametric data were appraised using the analysis of variance test, and non-parametric data were compared using the Mann-Whitney U test or Kruskal-Wallis test. A p-value <0.05 was considered statistically significant. The receiver operating characteristic (ROC) analysis was used to determine the cut-off values of APRI, NLR, and PLR. Analysis results were presented as median (quartile 1-quartile 3) for quantitative data and frequency (percentage) for categorical data. The significance level was considered to be p-values <0.050. The sample size was calculated by power analysis based on a previous study by Thalor et al. [18]. For the independent-sample t-test with a power of 2.1491714 and an α-value of 0.05, the power (1-β) was calculated to be 0.95 with 40 participants. Because the sample size in our study is larger than the calculated sample size, it is assumed that the power of the study is sufficient.

## Results

A total of 300 pregnant women were enrolled in this study. The study group included pregnant women with PE (n = 151). The mean age in the study was 32.0 ± 5.9 years, and the mean body mass index (BMI) was 25.5 ± 5.5 kg/m^2^. There was no significant difference in demographic data between the control group (n = 149) and the study group, which included healthy and normotensive pregnant women. Gestational age at hospitalization was significantly earlier in the hypertensive group than in the control group (33.43 (30.86-36.07) weeks/37.9 (37.0-38.9) weeks; p < 0.001). The PE group had significantly higher SBP and DBP values than the other group (p < 0.001) (Table 1).

**Table 1:** Comparison of the study groups by their general demographic and clinical characteristics. PE = preeclampsia; BMI = body mass index; SBP = systolic blood pressure; DBP = diastolic blood pressure; SD = standard deviation; IQR = interquartile range

Demographic data	Control (n = 149)	PE (n = 151)	P-value
Age (mean ± SD)	32.0 ± 6.6	32.0 ± 5.3	0.226
BMI (mean ± SD)	25.0 ± 4.1	26.0 ± 6.0	0.270
Gravida (median (IQR))	2 (2–3)	2 (1–3)	0.243
Gestational week at hospitalization (mean ± SD)	37.9 (37.0–38.9)	33.43 (30.86–36.07)	<0.001
SBP at hospitalization (median (IQR))	110.0 (100.0–120.0)	140.0 (140.0–150.0)	<0.001
DBP at hospitalization (median (IQR))	70.0 (60.0–80.0)	90.0 (90.0–100.0)	<0.001
Birth week (median (IQR))	39.0 (38.3–39.1)	37.0 (33.8–37.6)	<0.001

The median NLR, PLR, and APRI values obtained in venous blood samples collected during hospitalization (4.63 (3.61-6.73), 130.4 ± 55.7, and 0.28 (0.24-0.36), respectively) were not significantly different between the study groups (p = 0.639, p = 0.709, and p = 0.066, respectively) (Table 2). Cut-off values with the highest specificity and sensitivity were calculated using ROC analysis curves and predicted PE compared to the control group. The factors were considered univariate factors. When comparing the control group and the PE group using ROC analysis, none of the parameters could be used to predict PE (Table 3).

**Table 2:** Comparison of the study groups in terms of tested laboratory parameters at the time of diagnosis. PE = preeclampsia; NLR = neutrophil-to-lymphocyte ratio; PLR = platelet-to-lymphocyte ratio; APRI = aspartate aminotransferase-to-platelet ratio index

Parameters	Control (n = 149	PE (n = 151)	P-value
NLR (median)	4.55 (3.67–5.98)	4.63 (3.61–6.73)	0.639
PLR (median)	140.59 ± 112.82	130.4 ± 55.7	0.709
APRI (median)	0.27 (0.21–0.33)	0.28 (0.24–0.36)	0.066

**Table 3:** ROC results between the control and PE groups. NLR = neutrophil-to-lymphocyte ratio; PLR = platelet-to-lymphocyte ratio; APRI = aspartate aminotransferase-to-platelet ratio index; PE = preeclampsia; ROC = receiver operating characteristic; AUC = area under the curve; CI = confidence interval

Parameters	AUC (95% CI)	P-value
NLR	0.516 (0.45–0.581)	0.639
PLR	0.488 (0.422–0.553)	0.709
APRI	0.562 (0.497–0.627)	0.066

## Discussion

Although hypertensive disorders in pregnancy are defined as multifactorial and multisystemic, there are many unresolved questions regarding etiology. Previous studies have shown that the leukocyte count increases more in preeclamptic pregnancies than in normal pregnancies, and the main reason for this increase is the increase in neutrophils [7]. In addition, endothelial dysfunction in PE leads to increased platelet destruction in the peripheral circulation and, consequently, increased uncontrolled intravascular platelet activation. The APRI can be calculated using a formula that indicates liver function and is readily detectable in blood parameters related to inflammatory processes [12]. It is used in research as a marker of liver fibrosis and inflammation. Predicting PE in the first weeks of pregnancy is not yet possible, but the diagnostic value of the mentioned inflammatory markers is present. This has been shown in many diseases such as coronary artery disease, autoimmune diseases, and inflammatory diseases. This study aimed to determine whether a widely used, inexpensive, and simple test such as complete blood count has predictive value for this disease. We evaluated these three parameters in light of this information and concluded that they were not sufficient to predict PE. To evaluate our findings, the literature was reviewed in detail. The conclusion was that studies using PLR and NLR values to predict PE did not show a stable result.

A previous study reported that NLR and PLR values in blood samples before 20 weeks of gestation did not differ between the study and the control groups; however, in blood samples from the third trimester, higher NLR and lower PLR values were found in the PE group [19]. Gezer et al. [20] identified NLR as the strongest predictive variable in multiple regression analysis to predict PE. In the same study, the authors reported significantly higher NLR and PLR values in the PE group than in the control group [20]. Another study by Wang et al. [21] reported that NLR plays a predictive role in detecting PE and predicting disease severity [21]. One study reported elevated blood count markers in pregnant women with PE and concluded that NLR and PLR may be useful in predicting PE [16]. These studies show that these parameters are effective in predicting PE or determining the severity of the disease. However, in our study, we could not statistically demonstrate the difference between the study and control groups in the samples collected before treatment. On the other hand, many studies have reached similar conclusions to ours. One of these studies reported that NLR and PLR were not able to predict the progression from PE to severe PE [17]. In a study comparing healthy pregnant women with the PE group, it was reported that NLR and PLR could not be used to determine severe PE [22]. Kurtoğlu et al. [23] reported that the threshold for NLR was 4.48, with a sensitivity of 58% and a specificity of 63%, although it was not significantly associated with the severity of the disease [23]. In contrast, two studies investigated only PLR values. Accordingly, Kirbaş et al. [24] found a lower PLR compared with controls in the mild PE group, but the difference was not statistically significant. Surprisingly, PLR was higher in the severe PE group than in the mild PE and the healthy groups [24]. As this study was conducted in the first trimester, we thought that this result might lead to inconsistency. According to Yavuzcan et al. [17], PLR was lower in the severe PE group but not statistically significant. In another study, although there was no difference between the control and the PE groups in terms of NLR, the decrease in other parameters related to PLR and platelet levels was statistically significant [25]. Because of this pathophysiological step, several biomarkers have attracted attention in the second decade of the 21st century because they can be used to predict PE as early as the first trimester. Identification of patients at an increased risk of PE and early prediction of PE improve outcomes and allow prophylaxis with aspirin and early intervention. Studies have shown the hyperactivation of inflammatory cells and immunological responses of lymphocytes and neutrophils with the release of proinflammatory biomarkers leading to dysregulation of the endothelium. Low-grade inflammation is used to describe the condition defined by mildly elevated immune cell numbers and high proinflammatory protein levels in cases without evidence of inflammatory disease. The parameters in our study are readily available as markers of systemic inflammation from complete blood count and are used in the diagnosis and treatment of several inflammatory diseases. They are commonly used to predict complicated pregnancies. However, published findings on the association between preeclampsia and SIR markers remain controversial and conflicting. As mentioned earlier, some studies have shown that these markers are useful in predicting preeclampsia, while others have not.

To date, the number of studies on APRI and PE is limited. Sasmaz et al. [14] reported that at a cut-off value of 0.339, the APRI score predicted HELLP syndrome better than AST alone (sensitivity: 71.1%, specificity: 91.2%). Based on this result, the APRI score is an effective and useful method for predicting HELLP syndrome (not PE or severe PE) [14]. This interpretation for HELLP syndrome could not be made for PE in our study.

The results of our study should be interpreted considering the strengths and weaknesses. The main limitation is that these parameters were studied only in maternal peripheral blood. It is important to investigate cord blood and placental tissue samples in future studies. The strongest aspect of this study is that it was conducted in a tertiary care center with a relatively large sample size. In addition, the analysis of data from a single central laboratory eliminated device-dependent variability. This study is an important contribution to the literature by evaluating all parameters with a large number of cases. In addition, the literature has shed light on further investigation of APRI values in future PE studies.

## Conclusions

According to our study, the parameters controlling inflammatory processes have no clinical significance in assessing the risk of developing PE and predicting the severity of PE. Our results also highlight the difficulty of predicting hypertensive disease in pregnancy and show how costly this process has become. Prospective randomized controlled trials with a large number of cases and tissue samples are needed to provide more accurate information on this topic.
